# *Partitioning*, a Novel Approach to Mitigate the Risk and Impact of African Swine Fever in Affected Areas

**DOI:** 10.3389/fvets.2021.812876

**Published:** 2022-02-22

**Authors:** Solenne Costard, Andres M. Perez, Francisco J. Zagmutt, Jane G. Pouzou, Huybert Groenendaal

**Affiliations:** ^1^EpiX Analytics, Fort Collins, CO, United States; ^2^Center for Animal Health and Food Safety, College of Veterinary Medicine, University of Minnesota, Minneapolis, MN, United States

**Keywords:** African swine fever, disease control, biosecurity, surveillance, risk mitigation, swine depopulation, cost effectiveness

## Abstract

As African swine fever (ASF) continues to expand geographically, supplementary control strategies are needed to reduce disease risk and impact in affected areas. Full depopulation is central to current ASF control efforts, and its efficacy depends on surveillance and timely disease reporting, while resulting in large losses regardless of the producers' efforts to promptly detect, report, and contain the disease. This disconnect between prompt detection and reporting, and subsequent farm losses, can deter producers to invest in ASF detection and control. Alternative approaches are needed to incentivize individual producers to invest in early detection and reporting. We postulate that commercial swine farms may be effectively partitioned in separate units, or subpopulations, to which biosecurity, surveillance and control can be applied. The suggested Partitioning framework relies on three main components: 1. external and internal biosecurity to reduce the risk of ASF introduction and maintain separate subpopulations; 2. cost-effective on-farm ASF surveillance to enhance early detection; 3. response plans at the unit level, including culling of affected subpopulations, and demonstration of freedom from disease on the remaining ones. With such Partitioning approach, individual producers may reduce ASF risk on a farm and in the region, while also reducing ASF outbreak losses via targeted depopulation of affected units. It requires relevant legislation to incorporate the notion of within-farm subpopulations and provide a regulatory framework for targeted depopulation and substantiation of disease freedom. Its design should be tailored to fit individual farms. Partitioning can be an effective public-private partnership approach for ASF risk reduction. It should be driven by industry, as its benefits are accrued mainly by individual producers, but regulatory oversight is key to ensure proper implementation and avoid further disease spread. Partitioning's value is greatest for producers in ASF-affected regions, but ASF-free areas could also benefit from it for preparedness and early detection. It could also be adapted to other transboundary animal diseases and can be implemented as a stand-alone program or in conjunction with other efforts such as zoning and compartmentalization. Partitioning would contribute to the improved resilience and sustainability of the global pork industry and will benefit consumers and society through improved food security and animal welfare.

## Introduction

African swine fever (ASF) is one of the most devastating diseases of pigs, associated with high mortality rates and dramatic economic losses (1, 2). Its accelerating worldwide expansion over the last decade (3, 4) shows that current ASF control measures are insufficient to curb its spread. In most affected areas, official control programs are in place to contain and eradicate the disease, following the guidelines from the World Organization for Animal Health (OIE) (2, 5, 6). Generally, the basis of such ASF control and eradication programs is strict sanitary measures, including stamping-out policy and movement control (7). However, several barriers to effective implementation of these measures have hindered their efficacy (8, 9). In some endemic areas, the economic impact that these sanitary measures have on individual producers has resulted in practices such as the emergency sale of animals, which contribute to further disease spread (10). In addition, stamping-out policies typically rely on mass depopulation of farm animals (7), which increasingly raises public concerns about animal welfare, food security and sustainability (11–13).

Given the unprecedented spread of the disease, public concerns about animal culling, and in the absence of a “silver bullet” to control ASF, there is a need to consider additional control approaches based on public-private partnerships (PPP). Acknowledging this, the Food-and-Agriculture Organization (FAO) of the United Nations and the OIE launched in 2020 a new initiative for the global control of ASF (9) that highlights the need to facilitate business continuity, and mention zoning and compartmentalization as potential approaches to implement for this purpose. Such programs (14–16) require coordination between government and industry stakeholders, and require considerable organization, resources, and commitment at the industry-level (17), which can be challenging depending on the nature of the local pig production sector. The success of alternative approaches thus hinges on two aspects:

1. Creating cost-effective solutions that incentivize individual producers to invest in surveillance, early detection, and prompt reporting of ASF.
2. Implementing effective alternatives to full farm depopulation in case of an ASF outbreak, that is in line with appropriate supporting legislation.

In this paper, we propose an industry-led PPP initiative for ASF-affected areas to incentivize individual producers to invest in risk management measures, and thus enhance ASF prevention and control. Typically, commercial swine farms are organized in units (sites, barns) that could be “partitioned” into spatially and epidemiologically separate subpopulations. On this premise, we propose a Partitioning framework focused on on-farm activities for prevention, early detection, and control of ASF at the level of these subpopulations. This approach would require a legal basis and endorsement by the competent authority (CA). Here we describe the rationale for the Partitioning approach, outline its three main components and show its value to various stakeholders. We discuss how it can be implemented as a stand-alone effort or in complement to other programs, particularly in areas where ASF is present in domestic pig populations and with barriers to the implementation of current ASF control strategies.

## Assessment of Current Policy Options and Implications

ASF represents an increasing risk for pig producers worldwide. Current ASF and TADs control guidelines typically include culling of all animals in infected herds, cleaning and disinfection, movement control, and contact tracing (15, 18, 19). This strategy's effectiveness is highly dependent on early detection and prompt reporting by farmers. Yet, reporting of a suspected ASF case results in immediate economic costs to the producer due to a standstill until laboratory diagnosis is obtained, while earlier reporting of a suspected case does not decrease the eventual farm level losses—such as stamping out, loss of business, and time and resources needed to rebuild reproductive stock. Accordingly, individual producers often lack incentives to invest in on-farm ASF surveillance to detect infections earlier. This can result in delays in reporting of suspected ASF cases (20, 21) and thus hinders control by allowing for undetected disease spread before outbreak response is implemented (22). In areas where the disease has become endemic and/or where compensation after depopulation is insufficient, underreporting is common (1, 10, 23–25). Evidence suggest that in some settings, producers may instead proceed with their own disease control strategy without the oversight of CAs—for example, the emergency sale of animals not showing clinical signs combined with a partial depopulation strategy (10, 20, 26–28). Unfortunately, these practices likely contribute to further ASF circulation.

Governments and the OIE have established approaches to enhance early detection and mitigation of ASF risks, such as zoning or compartmentalization—which are particularly relevant for the purpose of international trade (29, 30). Both are referred to as “procedures implemented by a country […] to define subpopulations of distinct […] health status for the purpose of disease control or international trade.” Zones are defined based on geographical boundaries, whereas compartments are based on biosecurity practices and surveillance and require considerable coordination between many supply chain actors (17). Zoning and compartmentalization are however expensive to implement and maintain and may be impossible to achieve in many countries, as they require significant coordination between government, industry, and other stakeholders. Other initiatives, such as the Secure Pork Supply (SPS) (31), aim to ensure business continuity for US pork producers located in a control zone during an active ASF outbreak, provided that such producers meet certain enhanced biosecurity requirements and have no evidence of infection.

The abovementioned programs focus on reducing the economic impact of ASF from specific disease-free sectors of the industry. However, in line with laws and policies in places in most countries, an ASF outbreak in a participating farm typically results in its full depopulation regardless of the extent of infection on the farm. There is thus an opportunity for supplementing these existing strategies with an approach that incentivizes producers to invest in early detection and timely reporting in return for allowing for the depopulation of only affected units in the farm location. A growing number of experts support the development of these alternatives to mass depopulation (7, 8, 32). For example, in compartments of the South Africa ASF controlled areas, the extent of the depopulation in case of outbreak is determined on a case-by-case basis. The CA incentivizes biosecurity, surveillance and early reporting by not compensating for culled animals, and requiring 3 months after the last case before the ASF-free status can be restored: this way, compartment operators are motivated detect infected animals early and prevent on-farm spread to limit the number of animals culled, but will also want to make sure to properly eradicate infection on the farm to minimize time to recovery of their status (7, 33, 34).

This type of on-farm risk management is already a possibility for some non-notifiable and/or endemic diseases, such as porcine reproductive and respiratory syndrome (PPRS) in the U.S (35). In the absence of a regulatory framework, the surveillance and management of non-regulated “production diseases” (i.e., diseases which affect mortality, reproduction cycles, or growth) such as PPRS is left to the discretion of the producers and informed by the business and health management model of individual farms. Farmers will invest in prevention and control programs for such diseases if they consider that the potential decrease in disease costs is worth the investment.

## Actionable Recommendations

### Partitioning Framework

We propose a voluntary framework to improve detection and control of ASF (and by extension, other TADs) - such approach is possible if it can be shown that the investment is cost-effective for producers and that there is no increase in disease spread risk. This Partitioning framework for cost-effective risk management of ASF by individual producers relies on maintaining separate animal units via good external and internal biosecurity, conducting on-farm surveillance and early reporting, and in case of outbreak, coordinating with CAs for the safe targeted depopulation of affected units and demonstration of freedom in other units.

As explained earlier, a typical commercial swine farm can be “partitioned” into epidemiological units i.e., separate subpopulations. The OIE defines an epidemiological unit as a “group of animals (...) that share approximately the same likelihood of exposure to a pathogenic agent,” and an outbreak as the “occurrence of one or more cases in an epidemiological unit” (36). What constitutes an epidemiological unit may differ between diseases, affected species and/or type of production, and may correspond to “animals in a pen,” “herd” or “flock,” or even “village.” ASF prevention and control measures are applied at the outbreak level determined by that epidemiological unit, which varies between countries. For example, certain countries report ASF outbreaks in wildlife as “individual animals affected by the disease” (37, 38), whereas other countries refer to outbreaks in wildlife as “regions” in which the disease was identified (39, 40). Also, in countries with extensive backyard farming where free-range pigs coexist, the village or commune is considered the epidemiological unit (41–43), whereas in industrial swine production that definition is typically applied to the farm (15). Therefore, the definition of epidemiological unit to enforce outbreak control measures is flexible and linked to the concept of subpopulations' exposure and disease risk. As such, key factors in defining an epidemiological unit include the physical separation of animals (e.g., separate pens, rooms, barns) as well as management practices such as movement/flow of animals between units, movement of staff and visitors, and other internal biosecurity procedures. Any quarantine, surveillance, clinical inspection and/or testing before movement between groups of animals also contribute to what can be considered an epidemiological unit. A farm may represent, for example, common ownership, taxes, property, location of land dedicated to agricultural purposes. In commercial swine production, a farm may be partitioned in multiple epidemiological units, as the units of management are the barns and/or production units. For example, a farrow-to-finish farm can have separate buildings, locations, management practices and even personnel. Consequently, it could be divided into quarantine, gilt development unit (GDU), boar station, gestation unit and/or farrowing unit, growing unit, and finishing unit.

**Figure 1:**
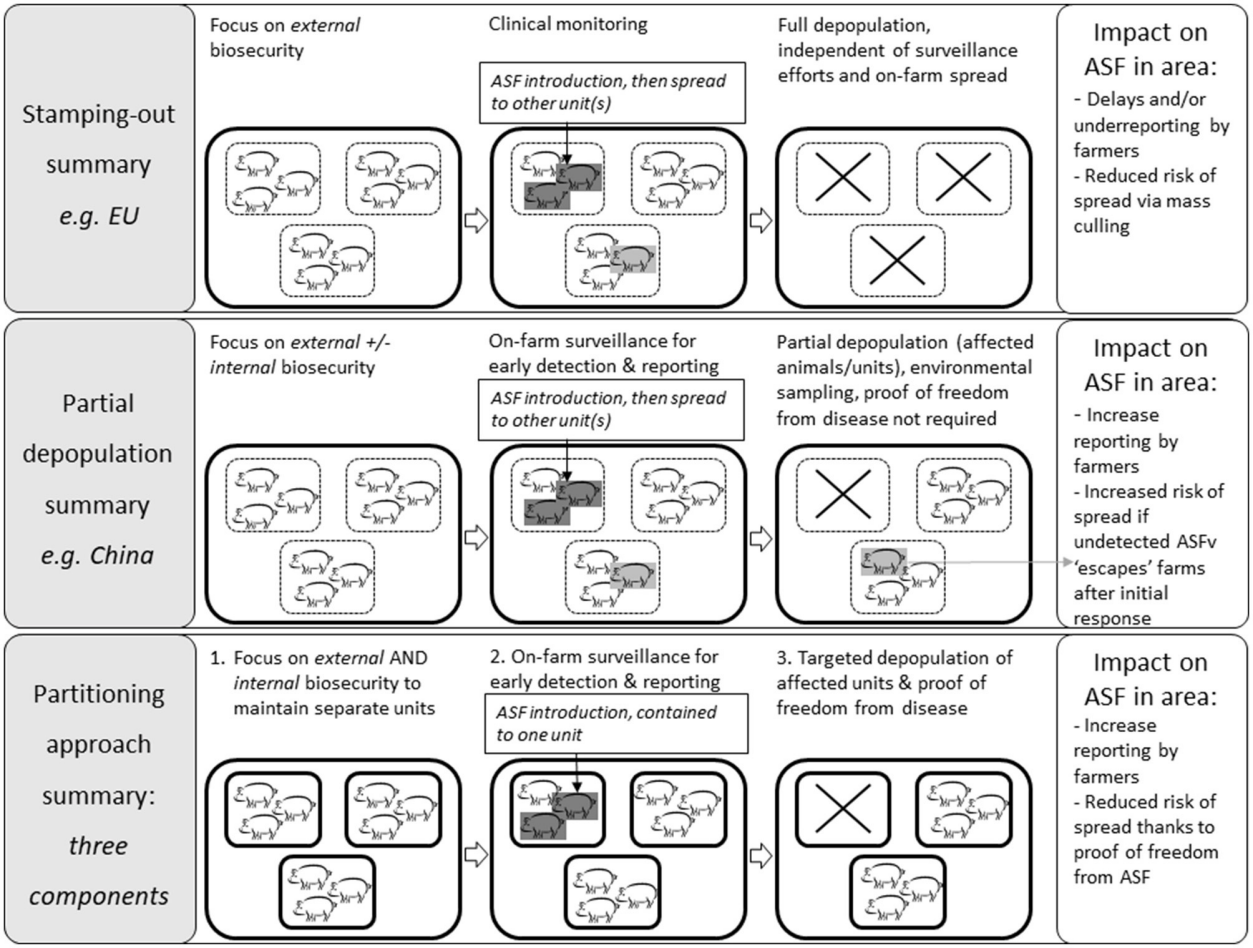
Comparison of the principles of the standard ASF control approach, partial depopulation, and Partitioning approach, using an example farm with three epidemiological units (e.g., three finishing barns).

Partial depopulation strategies for ASF are currently being implemented in ASF-affected areas in Asia (44, 45). In China for example, issues of underreporting and food security have motivated the CA to move away from mass depopulation. Some successes with partial depopulation on large commercial farms with good surveillance and biosecurity illustrates that it can be a feasible option (46, 47). On the other hand, the shortcomings of the “tooth extraction protocol” tested in Vietnam (48, 49) highlights that partial depopulation could result in further disease spread, when no additional measures limit on-farm disease spread and freedom from disease in remaining units is not established. Protocols in China require environmental testing of depopulated units and restocking only after all PCR tests are negative. Although important, this protocol does not ensure that the other units remain free of infection. For these reasons, the clear definition of within-farm epidemiological units is pivotal to a successful Partitioning approach for the swine industry. The CA would define what constitutes an epidemiological unit on a farm, and the corresponding process through which they would allow for targeted depopulation in case of outbreak i.e., a description of how control measures would be applied in infected unit(s) vs. disease-free ones, and the process through which freedom of disease would be established. In return, this regulatory clarity would increase swine producers' incentive to invest in additional internal biosecurity to separate epidemiological units, and surveillance to quickly detect and report any suspected cases to the CA. The overall result of Partitioning is to reduce losses in case of an ASF outbreak, both for the individual producers - by avoiding full farm depopulation - and for the industry and CA - by increasing early detection and reporting, reducing the risk of spread beyond the farm, and thus diminishing the overall epidemic size. To address potential challenges associated with partial depopulation, Partitioning encompasses additional efforts at the individual farm level (Figure 1):

1. Farm biosecurity: prevention of disease introduction and on-farm spread via external and internal biosecurity practices. This objective may be aided by decision-support tools to prioritize and harmonize efforts on individual farms.
2. Optimization of cost-effective on-farm surveillance, informed by modeling early detection, and prevention of disease spread between epidemiological units.
3. Preparedness for prompt response and control in case of disease introduction into farm unit(s). A response plan must be prepared and in line with government requirements for targeted depopulation, including notification of authorities and close collaboration for the implementation of safe targeted depopulation, as well as monitoring and demonstration of disease freedom on separate units. This monitoring plan can also be supported by modeling.

The Partitioning framework contains components that should be part of any ASF prevention and control strategy (e.g., external biosecurity, reporting of suspected cases to CA) as well as items more specific to the Partitioning framework which we further discuss below.

#### Farm Biosecurity

Biosecurity comprises procedures and practices both to prevent disease introduction into a farm (external biosecurity), and to reduce the risk of disease transmission between areas of a farm (internal biosecurity). Most existing biosecurity tools' emphasis is on external biosecurity (50). While external biosecurity is fundamental to reducing the risk of ASF introduction, Partitioning also requires high internal biosecurity to maintain separate epidemiological unit(s) and contain a disease outbreak to the unit(s) where it got introduced initially (50, 51).

Many biosecurity guidance and scoring tools exist but they are not always well adopted, and/or compliance by staff is not always observed. They can indeed be cumbersome, and the efficacy of separate biosecurity measures is not well measured (51–54). For Partitioning, we propose a biosecurity approach that focuses on harmonization, and prioritization of improvement recommendations (manuscript in preparation). Rather than relying on an overall biosecurity score, this approach would assess the consistency of biosecurity measures across farm management areas and identify the weakest practices(s) for improvement efforts. Ultimately, the weakest biosecurity practices influence the separation between epidemiological units, and therefore the type Partitioning achievable on a farm. For example, some producers may only partition their farm in sow vs. grow-to-finish units, while others may be able to further subdivide sow sites in gilt development units, farrowing units and gestation units thanks to very strict internal biosecurity. This could also change over time as biosecurity improvements are implemented.

In addition, biosecurity measured by an overall score fails to distinguish a farm that performs well in almost all aspects but very poorly on a few biosecurity measures from a farm that performs fairly well across all biosecurity aspects. Ultimately, the weakest links can affect the overall biosecurity (both external and internal) of a farm (55), hence the rationale for consistency in farm biosecurity levels, and the focus on addressing these weak links before investing in improving other biosecurity aspects.

Because understanding the reasons for control measures increases compliance (56), the approach would also highlight the rationale and possible efficacy of recommended measures and priorities. This will foster practice-based knowledge and encourage producers' “buy-in” and compliance, as compared with a strictly regulatory imposition of biosecurity standards. In addition, since every producer would benefit from better biosecurity and surveillance at every farm outside of their own, knowledge exchange would also be stimulated.

One could question the credibility of internal biosecurity in case of an ASF outbreak because the external biosecurity failed. However, there are many external biosecurity aspects that also involve other stakeholders who may not have the same protocols or incentives for compliance (suppliers, visitors, etc.), possibly resulting in biosecurity breaches. In contrast, internal biosecurity can be more tightly controlled as it relies on on-farm procedures for the movement of animals, people, and equipment between production units. Finally, other activities such as on-farm surveillance and demonstrating disease freedom following an outbreak will complement internal biosecurity measures to document effective Partitioning of units, as discussed hereafter.

#### Optimization of Cost-Effective On-Farm Surveillance

In Partitioning, the objective of on-farm ASF surveillance is to improve time to ASF detection and decrease the probability of further spread to additional units on the farm. Commercial swine farms typically conduct clinical monitoring for several targeted diseases, and additional surveillance such as ASF diagnostic tests may also be in place in ASF-affected areas. But under most ASF control programs, early detection and reporting results in full farm depopulation. Early detection may even be associated with additional losses for the individual producer: it implies contacting the CA as soon as suspect—and typically unspecific—signs are noticed and being imposed a standstill while waiting for laboratory results. Some of these suspicions may be caused by other conditions (false alerts, or false positives)—even more so as they rely on early and unspecific signs—and in such cases, activities resume once the farm is confirmed negative. As pork production relies on a flow of animals between buildings, from the gestation unit to the farrowing unit to grower and finisher barns before shipping to slaughterhouses, a standstill results in units being backed up and crowded, and a disruption of the gestation and farrowing units. The hog market crisis earlier in the Covid-19 pandemic (57, 58) provides an example of the effects of such pork production disruptions. In summary, producers currently have an incentive to wait until they are confident that disease is occurring in their herd before reporting their concerns as required by the CA.

With Partitioning, an early detection of ASF incursion results in the implementation of control measures before the disease spreads to other units on the farm and thus the reduction of losses thanks to targeted depopulation—providing the other units are proven to remain free of disease. Successfully containing the infection can also be easier when it is limited to a subset of the farm.

Although early detection can reduce outbreak costs—animal losses, cleaning, and disinfection, potential loss of trade partners, time until recovery, etc., conducting additional active on-farm surveillance for early detection as part of Partitioning also has direct costs: staff time to conduct regular clinical monitoring, biological samples, diagnostic kits, and laboratory analysis. In addition, the probability of false positives will increase with additional surveillance, with subsequent costs. On-farm surveillance for Partitioning relies on clinical signs and/or regular sampling and testing for preclinical detection. ASF symptoms are unspecific, especially those during the early phases of an ASF outbreak or in CSF-endemic areas, and although ASF diagnostic tests have good performance, they are imperfect. For example, due to their lower specificity, the use of antigen detection ELISA and pen-side tests or isothermal assays for preclinical detection would lead to false positives (59–64). Also, the probability of a false positive may increase if criteria for suspecting a case are changed and become extremely sensitive, e.g., if the alarm is raised as soon as any potential sign of disease (e.g., loss of appetite, depression, or fever) is noticed on a single animal. This is analogous to a diagnostic test with a high sensitivity but low specificity. When a suspicion is reported in such instances, standstill costs are incurred until the laboratory results are obtained to clear or confirm the outbreak. These complex tradeoffs between the various costs and benefits of on-farm surveillance and early detection motivated us to develop a stochastic modeling tool that optimizes the return on investment of on-farm surveillance for Partitioning (65). The tool considers factors specific to a given farm, such as the number of units, the relative number and economic value of the animals, and the producer's risk tolerance. It also accounts for the disease context of the farm location: affected area or disease-free zone, ASF incidence, policy for culling (e.g., if a farrow-to-finish farm is found to only have a farrowing room infected, either the full sow unit or only the farrowing unit could be culled) and CA indemnification (value per animal, time to compensation, limited budget for compensation at a certain administrative level, etc.). Finally, it explicitly models within-unit ASF spread dynamics and diagnostic options, including clinical monitoring, type of tests and their performance, costs, and time to results. By considering farm management, economic aspects, disease dynamics and diagnostics, the tool supports the development of a tailored on-farm surveillance regime that enhance early detection while keeping surveillance costs acceptable to the individual producer.

#### Preparedness and Response Plan

A plan of action in case of ASF suspicion must be prepared in compliance with relevant CA regulations. Its implementation must be coordinated with the CAs, who are ultimately responsible for overseeing the official control program (66).

The response plan needs to be precise and actionable so that it can be activated immediately following a suspected case. The definition of an ASF suspect should be pre-defined, and once notified, a farm-level standstill protocol activated until the laboratory confirmation is available. If the outbreak is confirmed, depopulation of the infected epidemiological units will be carried out as required by the CA. This action will be complemented by cleaning and disinfection, reinforced biosecurity, and on-farm surveillance to mitigate the risk that the disease spreads to other units. In addition, substantiation of freedom from disease will be conducted in the remaining units before the standstill can be lifted for these units. CAs would specify requirements to achieve a desired target minimum detection level in the epidemiological units being monitored and specify the diagnostic test(s) that can be used for this purpose. Modeling can support the development of such response plans, especially for designing cost-effective monitoring plans during the standstill period and for demonstrating freedom of disease following an outbreak.

This third component is the one for which appropriate legislation and a clear regulatory framework is required. The definition of what constitutes an epidemiological unit needs to be clear, as well as the requirements for reporting, and procedures to determine the extent of on-farm depopulation as well as to demonstrate disease freedom on other units, etc. As mentioned previously, this regulatory clarity will allow producers to make an informed decision on whether to invest in Partitioning.

### Partitioning for Different Stakeholders

Partitioning should largely be funded by individual producers, but requires a clear regulatory framework in line with supporting legislation. Regulatory certainty gives individual producers a financial motivation to invest in measures to meet such requirements, in exchange for the possibility to reduce their losses. Partitioning is therefore a good example of a PPP effort, in which the public and private sectors work together to efficiently reduce disease risk (Table 1). We believe that giving individual producers additional rewards for ASF risk mitigation will result in an efficient use of resources and enhance and complement national or industry-wide approaches such as national passive surveillance programs. The high biosecurity and active on-farm surveillance will not only benefit farms participating to the Partitioning approach but also other pig producers in the same area, while the requirement to demonstrate disease freedom on remaining units in case of outbreak will mitigate the risk of further disease spread. Farm-level early detection of ASF in an affected situation may be achieved by regulatory requirements but providing economic incentives to producers to do so will increase the cost-effectiveness of surveillance: producers have the most to lose due to outbreaks, while they have the best knowledge of their herd-specific economic and health situation. While it will not always be possible to only depopulate only part of the herd in case of outbreak on a farm implementing Partitioning, this regulatory clarity is essential to establish rules for the extent of farm depopulation. Partitioning relies on the CA's capabilities to balance the requirements for targeted depopulation and substantiation of disease freedom at the farm level to mitigate the risk of disease at the industry level vs. the needs to adapt surveillance, detection, and control efforts to the unique circumstances of the local pork industry. We also emphasize that the exact Partitioning regulations and implementation procedures will differ considerably between regions and countries (7, 8), given differences in operational and economic circumstances.

**Table 1:** Anticipated costs and benefits of partitioning for different stakeholders.

**Stakeholder**	**Costs**	**Benefits**
Individual producer	On-farm biosecurity, surveillance, and response preparedness to meet requirements established by competent authority	Reduction of ASF risk: • Lower risk of outbreak • Reduction of losses in case of outbreak (targeted depopulation, time to recovery)
Pork industry	Communication and training on Partitioning and its components: • Biosecurity and on-farm surveillance • Clinical monitoring vs. laboratory diagnosis • Response preparedness, etc.	Reduction of ASF risk: • Early detection and response • Lower risk of spread Potential step toward more formal, industry-wide efforts (e.g., compartmentalization, certification, etc.) Reduced financial risk to industry stakeholders (banks/lenders, insurance companies, etc.)
Competent authority	Develop regulatory framework for targeted depopulation: • Biosecurity and/or surveillance requirements, • Outbreak response, demonstration of disease freedom on farm units, etc. Need to adapt regulatory framework to local context Oversight and close collaboration with producers for outbreak response	Reduction of ASF risk: • Increase sensitivity of surveillance • Early detection and response • Lower cost of surveillance • Lower risk of spread Lower cost of outbreak control: • Decrease of number of animals culled • Lower rendering effort • Lower level of compensation of producers' losses, etc.
Society	Commitment to follow supporting regulation to mitigate the risk for disease spread	Reduced impact of ASF: • Targeted rather than mass culling • Lower environmental impact of rendering • Lower animal welfare impact and better general population acceptance • Lower mental health toll of depopulation (producers, animal health practitioners, etc.) • Better food security (more stable supply chain and pork price) and industry sustainability

The following aspects should be considered when designing a Partitioning program:

1. *Tailoring Partitioning to individual situations:* the higher the sensitivity of on-farm surveillance, the earlier the detection, but also the higher the costs for individual producers, as discussed previously. On-farm surveillance should account for the large diversity of production systems and will thus need to be customized to each farm to be cost-effective. Partitioning protocols should also be tailored to consider disease incidence, farm type, structure and management, access to laboratory diagnostics, economic situation, potential for investment, attitude toward risk, etc. For example, producers may decide to go beyond the minimum requirements set by the CA, depending on their individual cost-benefits, appetite for risk, and investment capacity. Also, not all swine farms, nor units on a swine farm are equal, and options for control measures to prevent spread between units will vary depending on the type of units considered. For instance, it is generally easier to restrict movements in growing and finishing units than it is in sow units, where animals are regularly moved back and forth between gestation and farrowing units.
2. *Requirements to establish freedom from disease following an outbreak:* while ASF-affected units will be depopulated, other units will have to be shown to be free of infection following standards from the CA, to avoid further disease spread. Such standards need to consider the balance between the confidence in the disease-free status of epidemiological units and its cost: setting the monitoring standards too low could result in ASF spreading undetected to other units or farms, with a negative impact on all stakeholders, but setting them too high will increase the costs of Partitioning and reduce the net economic incentive for producers to invest in Partitioning.
3. *Balancing the costs and benefits to the CA:* Establishing requirements for Partitioning and safe targeted depopulation with demonstration of disease freedom will result in additional rules and procedures for the CA, and implementation will involve coordination with producers. Conversely, the widespread adoption of Partitioning can result in a reduction of ASF circulation overall, and better allocation of the CAs resources. The burden of this additional complexity would also have to be balanced against the benefits of Partitioning to the various stakeholders (Table 1).

Overall, we foresee that participating producers will apply to qualify for Partitioning, based on requirements set by the CA. This application will include a description of biosecurity efforts implemented to maintain separate epidemiological units, protocols for on-farm surveillance, and a detailed response plan aligned to regulatory requirements. Although producers may get external help for the design and/or implementation of on-farm protocols, we do not anticipate mandatory auditing by a third party. This is what we consider an “outcome-based approach,” because the extent of depopulation by the CA in case of an ASF outbreak will depend on whether units can be shown to be and remain free from ASF, rather than on the biosecurity and surveillance processes in place on the farm. Ultimately, it is in the interest of the producers to implement these processes properly to have a chance to prevent on-farm ASF spread and qualify for targeted depopulation. However, depending on the local context, the objective of the ASF program, the resources of the CAs, and the partnership between industry and government, a more “process-based” Partitioning approach involving auditing of farm practices may be considered. Regardless, producers will need to collaborate closely with the CA for the implementation of the response plan in case of ASF outbreak.

### Partitioning and Other ASF Control Efforts

Partitioning aims to reduce disease incidence in affected areas where strategies based on stamping-out policies have not brought ASF under control, and/or where the application of partial depopulation without substantiation of disease freedom may have led to further disease spread (Figure 1).

Partitioning could be implemented on its own, or be used as part of a broader strategy, in complement with existing approaches (Table 2). For example, it could be adopted in coordination with efforts such as the US SPS program, US Swine Health Improvement Plan (US SHIP) (67), or be incorporated into compartmentalization – e.g., following South Africa's model for ASF controlled areas, but replacing the 3-month waiting period after an outbreak by the farm-level substantiation of disease freedom.

**Table 2:** Overview of existing tools for FAD risk reduction and business continuity, together with Partitioning (CA: competent authority).

	**Zoning**	**Compartmentalization**	**US Secure Pork Supply**	**Partitioning**
Key objectives	Establish and maintain a disease-free sub-population within a territory, based on geographical limits	Establish and maintain a disease-free sub-population within a territory, based on biosecurity measures	Encourage outbreak preparedness, and provide option for animal movements in control area of a FAD outbreak	Reduce risk of on-farm introduction, maintain separate sub-populations on farm, incentivize early detection and reporting
Scope of business continuity	All animals in ASF-free area	Animals in ASF-free compartment	Participating farms in control area and with no evidence of disease	Participating farms experiencing an outbreak
Business continuity benefits	Movement of animals/products, national and international trade	Movement of animals/products, national and international trade	Movement of animals/products, national trade	Potential for lesser losses and reduced time of movement control
Role of CA (following program establishment)	Movement control and surveillance	Verification of compartment: biosecurity, surveillance, contingency plan	Verification of requirements: biosecurity, surveillance, movement records) Issue of movement permit during outbreak	Verification of requirements: surveillance for rapid detection and disease freedom Oversight of outbreak response
Who bears most of the cost	CA	Industry (with CA oversight)	CA	Industry (with CA oversight)

Partitioning is also relevant in affected areas where strategies such as zoning or compartmentalization are not yet achievable due to limited resources of the CA and/or the nature of the local pig production sector. Indeed, because Partitioning focuses on risk management at the individual farm level, it is expected to require less coordination between industry stakeholders and may thus be easier to achieve. In such situations, Partitioning concepts could serve as a stepping stone to compartmentalization.

Generally, the value and applicability of Partitioning is driven by the following factors:

1. Partitioning is of higher value in areas with higher risk of ASF infection in domestic pigs. This is because additional surveillance gets more cost-effective as the risk of outbreak and subsequent full depopulation increases. Partitioning is particularly relevant in regions such as Asia (e.g., Vietnam, China), Eastern and southern Africa (e.g., Uganda, South Africa), Russian Federation, and the Caribbean (e.g., Dominican Republic). However, with the increasing spread of ASF worldwide, Partitioning can also have value in free areas under increased ASF risk of introduction, for preparedness and to increase the chance of early detection when ASF is introduced into a new area, before it further spreads. A good example for this are protection zones recognized by OIE (29), such as Puerto Rico given its proximity to the island of Hispaniola which is experiencing an ASF epidemic (68).
2. Fewer CA resources for compensation of producer's losses, including those from culled animals, result in greater economic value of Partitioning to individual producers.
3. The value of Partitioning also increases where the overall structure of the pork industry is complex and/or less centrally organized, making it difficult to initially meet the requirements for a compartmentalization effort or other certification programs. Partitioning can be a step toward these larger efforts.
4. For producers who are already part of (an)other control effort(s), the marginal benefits of Partitioning may be lower, but the investments and costs needed to join will likely also be lower.
5. Partitioning is more valuable for farms with larger numbers of separate production units, and smaller epidemiological units relative to the overall size of the farm. This is because the potential reduction in a producer's loss compared to full depopulation is greater compared to farms where animals are housed in a few units. However, such benefits of smaller epidemiological units would need to be weighed against the higher costs of internal biosecurity and on-farm surveillance that this may cause.

## Discussion

We propose Partitioning as a cost-effective framework to help producers reduce their risk of ASF introduction as well as their losses in case of ASF outbreak. If widely adopted, it can also reduce the overall size of an ASF epidemic in a country or region. Given the continued expansion of ASF worldwide and the challenges with existing control efforts, there is a need for alternative disease management and control approaches that allow producers to better handle disease risk and impact in an endemic area or during an active epidemic. Partitioning would be particularly relevant in areas where ASF is present in domestic pig populations, and where there are barriers to the implementation of existing control measures relying on full depopulation. In addition, as mass depopulation is becoming increasingly unacceptable to the public, Partitioning can also help address animal welfare, sustainability, and food security concerns.

While this paper focused on ASF, the Partitioning framework can be used to manage other TADs. As biosecurity measures and required investments are similar for other TADs, applying the Partitioning framework to multiple TADs would result in a greater return on investment for producers.

Partitioning requires a legal basis so that CAs can provide a regulatory framework for the recognition of within-farm epidemiological units and requirements for targeted depopulation and substantiation of disease freedom. There is a need for organizations setting international standards as well as CAs to consider such notions and discuss guiding principles for their implementation. As illustrated by the change from mass culling to partial depopulation in China, this is becoming urgent given the ongoing global spread of ASF, the limitations in the implementation of current control strategies relying on stamping-out policies, and public concern regarding the welfare and sustainability issues of mass culling (13).

In many ASF-affected countries, producers have started implementing partial depopulation (20, 26–28). However, this is typically done without the endorsement of CAs and is sometimes combined with the emergency sale of animals without symptoms. This is problematic, as such actions contribute to disease spread (10). This highlights the need for clear rules for partial depopulation, including the requirement to demonstrate disease freedom in other units of the farm, and for coordination between government and producers for the implementation of the response plan in case of outbreak.

With the growing demand for animal protein and increasing need for sustainability in food systems, new effective disease control approaches that result in less waste due to mass depopulation are needed. Partitioning offers an approach that can be as effective as current control methods while also resulting in an increased sustainability of the pork industry and better public perception of outbreak management.

Better on-farm risk management, including on-farm surveillance and early reporting, will increase when the economic incentives of individual producers are aligned with that of competent authorities (69, 70), such that individual producers are better off when an outbreak gets reported early. Partitioning is a way to introduce such economic motivations for individual producers. This is relevant both early, immediately after the introduction of ASF into a country or region, as well as when ASF has become endemic.

While Partitioning may initially be of most interest to larger commercial swine farms that are organized in separate production units, the concept of cost-effective disease risk management associated with Partitioning can also be used for smaller producers. For example, it could provide them with a better economic incentive to invest in measures to separate animals of higher economic value from the rest of the herd. In addition, any additional investment by larger producers in early surveillance and reporting will also, indirectly, benefit smaller producers through ASF spread reduction in the region. The use of Partitioning by larger farm will also likely result in improvements in technology and practices, such that it will become more affordable and accessible over time, for increasingly smaller producers.

This paper describes the overall concept of Partitioning, and more detailed descriptions of its main components will be provided in separate articles. Biosecurity is key to the prevention of ASF risk, but the design and implementation of a biosecurity plan can be challenging for individual producers. In a forthcoming paper, we describe an approach for helping producers prioritize biosecurity measures and align both external and internal biosecurity as part of the Partitioning framework. Second, what constitutes a cost-effective on-farm ASF surveillance program will vary depending on multiple factors, including the farm considered and its epidemiological context. Other factors that need to be considered include the costs of testing, the overall sensitivity and specificity of the surveillance, benefits of finding a true-positive ASF case, as well as costs due to false positive or false negative results. Modeling to support the design of on-farm surveillance for Partitioning is being conducted. Early results were reported by Pouzou (65), but more will be provided in a forthcoming publication.

We hope that this paper will provide a basis for further research and discussions and contribute to the development and implementation of alternative strategies and tools to reduce ASF risk, such as the proposed Partitioning framework.

## Author Contributions

SC, HG, and FZ designed the project and conceived the presented idea. SC and HG wrote the first version of the manuscript. All authors provided critical feedback and helped shape the research and manuscript.

## Funding

This work was supported by Section 108 Foreign Currency Program (Federal Award Identification Number 108-2019-05) from the USDA FAS.

## Conflict of Interest

SC, FZ, JP, and HG are employed by EpiX Analytics LLC. All authors declare that the research was conducted in the absence of any commercial or financial relationships that could be construed as a potential conflict of interest.

## Publisher's Note

All claims expressed in this article are solely those of the authors and do not necessarily represent those of their affiliated organizations, or those of the publisher, the editors and the reviewers. Any product that may be evaluated in this article, or claim that may be made by its manufacturer, is not guaranteed or endorsed by the publisher.
